# Association between relative fat mass (RFM) and gallstones in adults: Results from NHANES 2017 to 2020

**DOI:** 10.1097/MD.0000000000044996

**Published:** 2025-10-10

**Authors:** Wenshan Zhu, Xiaofeng Deng, Yucai Jiang

**Affiliations:** a Department of Pharmacy, Ji’an Central People’s Hospital, Ji’an, Jiangxi Province, China; b Department of Neonatology, The Maternity and Child Health Hospital of Ji’an, Ji’an, Jiangxi Province, China; c Department of Pharmacy, Affiliated Hospital of Putian University, Putian, Fujian Province, China.

**Keywords:** body mass index, cross-sectional study, gallstones, NHANES, relative fat mass, waist circumference

## Abstract

Gallstones have been linked to obesity, and relative fat mass (RFM) is an emerging, simple, and low-cost obesity index that estimates body fat percentage without requiring body weight measurements. This study aims to investigate the relationship between RFM and the prevalence of gallstones among adults in the United States. We analyzed the 2017 to 2020 National Health and Nutrition Examination Survey (NHANES) data to assess associations between gallstones and 3 adiposity indices (body mass index (BMI), waist circumference (WC), and RFM) using descriptive statistics, multivariable regression, and subgroup analyses. Nonlinear relationships were examined using restricted cubic spline (RCS) and threshold effect analyses. Receiver operating characteristic (ROC) curves were generated for RFM, BMI, and WC in adjusted models to compare their predictive power. Of the 5340 participants aged 20 years and older included in the study, 580 had gallstones. In fully adjusted models, all obesity indices were associated with gallstones (*P* < .0001). For every 1 SD increase in WC, BMI, and RFM, the rate of gallstones increases by 3%, 5% and 9%, respectively. Participants in the highest quartile of RFM had a 381% higher prevalence than the reference group (*P* for trend <.0001). This association was particularly stronger in populations aged 20 to 60, with significant interactions. RCS showed a nonlinear association between RFM and gallstones (*P* for non-linear <.01). Further threshold effect analysis indicated that the inflection point for RFM was 35.98. ROC curve comparisons demonstrated superior discriminative ability of the RFM-based model (AUC = 0.751) over both BMI-based (AUC = 0.724) and WC-based models (AUC = 0.715), indicating enhanced predictive accuracy for gallstones. The high level of RFM was significantly associated with higher odds of gallstone prevalence, particularly in those under 60 years old. Furthermore, RFM has better predictive ability for gallstones compared to BMI and WC. These findings suggested that RFM could be a superior tool for gallstone risk assessment in clinical practice.

## 1. Introduction

Gallstones are a common digestive disorder marked by the formation of stones in the gallbladder or bile ducts, primarily due to high levels of cholesterol and bilirubin in the bile. The condition affects approximately 10 to 20% of adults globally,^[1–4]^ with significant variations among ethnic groups, up to 70% in American Indians and 10 to 15% in Caucasians,^[5,6]^ while Asian populations report lower prevalence rates.^[7,8]^ Although gallstones are generally benign, they can lead to serious complications such as acute cholecystitis, cholangitis, and pancreatitis if not addressed promptly.^[9]^ Despite previous studies identifying various risk factors associated with gallstone formation, there remains an absence of dependable clinical markers to effectively predict the likelihood of developing gallstones or to guide preventive measures. It should be noted that gallstone assessment in population-based studies often relies on self-reported data, which may introduce detection bias since clinical ultrasound confirmation is typically performed only in symptomatic individuals. Consequently, such datasets likely overrepresent symptomatic cases while potentially missing asymptomatic gallstones. Nevertheless, despite these limitations, self-reported gallstone data can still provide valuable insights for gallstone-related research.^[10]^

Obesity is recognized as a multifaceted metabolic disorder,^[11]^ and its prevalence has surged significantly in recent decades, reaching alarming proportions.^[12]^ Currently, nearly one-third of the world’s population is classified as obese, highlighting a critical public health challenge that demands urgent attention,^[13]^ and the fact that the obesity rate among U.S. adults is now a staggering 40%.^[14]^ Obesity has been shown to be strongly associated with various diseases, including but not limited to cardiovascular disease, hypertension, diabetes, hyperlipidemia, stroke, cancer, psychiatric disorders, and sexual and gynecological problems.^[15–18]^ Additionally, obesity is strongly associated with the development of gallstones.^[19]^ Notably, rapid weight loss, such as after bariatric surgery, can also provoke gallstone formation due to excessive cholesterol hypersaturation in bile.^[20]^ Body mass index (BMI) and waist circumference (WC), which are widely used indicators of obesity, do not effectively distinguish between muscle and fat mass.^[21]^ Investigators have uncovered a new indicator of obesity, relative fat mass (RFM), which includes WC and height to better assess fat mass.^[22]^ When assessing an individual’s total body fat percentage, RFM provides a more accurate measurement compared to BMI and WC. Importantly, growing evidence demonstrates that this novel metric is not only superior in assessing adiposity but also clinically significant, as multiple studies have shown RFM to be strongly associated with various obesity-related diseases, including nonalcoholic fatty liver disease, metabolic syndrome, cardiovascular disease, and gallstones.^[23–28]^

The association between RFM and gallstones remains to be explored. Our research utilizes data from National Health and Nutrition Examination Survey (NHANES) to investigate the association between RFM and gallstones, aiming to provide more practical clinical indicators for predicting the development of gallstone disease.

## 2. Methods

### 2.1. Survey description

The authors obtained data from NHANES, a population-based national cross-sectional study conducted by the National Center for Health Statistics to investigate the nutrition and health status of Americans. The study used a complex multi-stage layered probability sampling method, which was conducted every 2 years; thus, the samples were representative.

The National Center for Health Statistics Research Ethics Review Board approved all NHANES research programs and obtained written informed consent from all survey participants or their parents and/or guardians. All detailed NHANES research designs and data are publicly available at www.cdc.gov/nchs/nhanes/.

### 2.2. Research population

**Figure 1.**
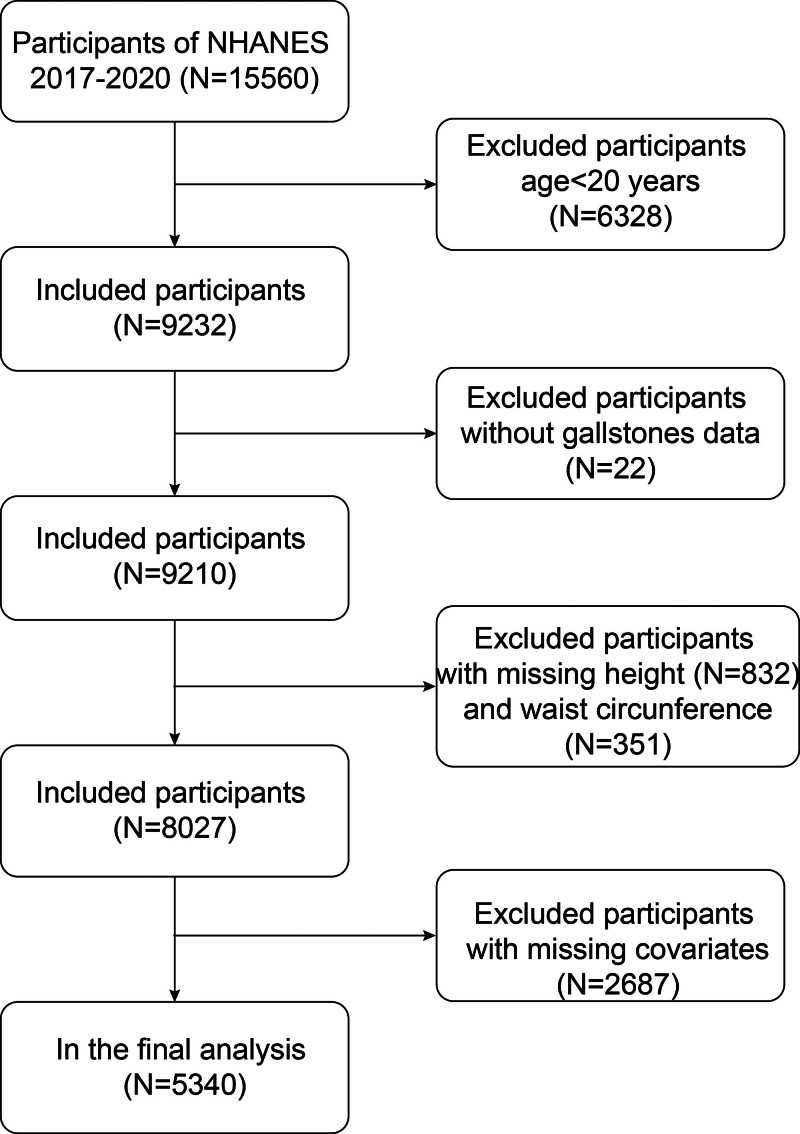
Flow chart of participants selection from the NHANES 2017–2020. NHANES = National Health and Nutrition Examination Survey.

Owing to the availability of gallstone information in the questionnaire data, this study only used NHANES data during January 2017 through March 2020 to explore the association between RFM and gallstones. From the initial 15,560 participants, we applied the following inclusion criteria: age ≥ 20 years; available data on gallstone status; valid RFM measurements; and complete covariate information. Participants were excluded if they: were aged < 20 years; had missing gallstone data; had missing RFM values; or had incomplete covariate data. Specific results are shown in the flowchart in Figure 1. Overall, the study eventually included a total of 5340 cases, including 580 cases with a self-reported history of gallstones.

### 2.3. Definition of relative fat mass and gallstones

The RFM is calculated by WC, height and sex. RFM =64 − (20 × height/WC) + (12 × sex), sex = 1 for women and 0 for men.^[22]^ Height and WC were calculated in cm in the formulas. In the analysis, RFM is viewed as a continuous variable, and the participants are grouped according to the RFM quartiles for further analysis.

We determined whether participants had gallstones based on the results of the questionnaire “Has a doctor or other health professional ever told that you had gallstones.” participants who answered “yes” were classified as having gallstones, while those who said “no” were categorized as not having gallstones.

In our study, the RFM was designed as the exposure variable, and the gallstones were treated as outcome variables.

### 2.4. Covariates

Covariates in our investigation included sex (male/female), age (year), race (Mexican American/other Hispanic/non-Hispanic White/non-Hispanic Black/other race), education level (<9th grade, 9–11th grade, High school graduate, Some college or AA degree, College graduate or above), poverty-to-income ratio, total Cholesterol (mg/dL), high density lipoprotein (mg/dL), hypertension (yes/no), and diabetes (yes/no), asthma (yes/no), and dietary intake factors, which comprised total energy intake (kcal), fat intake (g), sugar intake (g), water intake (g), dietary fiber intake (g), caffeine intake (g), and alcohol intake (g). All participants were eligible for 2 24-hour dietary recalls, and the average consumption of the 2 recalls was used in our study. Furthermore, anemia was defined according to WHO criteria as hemoglobin levels <13 g/dL in men and <12 g/dL in non-pregnant women.^[29]^ Smoking status was defined as having consumed more than 100 cigarettes in the past. Appropriate recreational activities were defined as participants who answered “Yes” to engaging in moderate-intensity sports, fitness, or recreational activities (e.g., brisk walking, bicycling) for ≥10 minutes continuously in a typical week, while “No” respondents were classified as inactive. All detailed measurement processes of these variables are publicly available at www.cdc.gov/nchs/nhanes/.

### 2.5. Statistical analysis

All statistical analyses were conducted according to Centers for Disease Control and Prevention guidelines using appropriate NHANES sampling weights and accounted for complex multistage cluster surveys.^[30,31]^ Descriptive statistics were used to explore general characteristics of the gallstones and non-gallstones populations in the United States. Continuous variables are expressed as the means with standard deviations, and categorical parameters were expressed as proportions. Logistic regression models were used to explore the association between BMI, WC, RFM, and gallstones. Furthermore, we divided RFM into quartiles (Q1: 10.09–30.09, Q2: 30.09–35.88, Q3: 35.88–43.88, Q4: 43.89–58.24), with the lowest quartile as the reference category. In Model 1, no covariates were adjusted. Model 2 was adjusted for sex, age, and race, and Model 3 was adjusted for all covariates. Age was first transformed into a categorical variable with 3 age groups: aged 20 to 39 years, aged 40 to 59 years, and ≥60 years. Subgroup analyses and interactions stratified by age, sex, race, smoking status, hypertension, and diabetes. The study constructed 3 composite models by integrating adiposity indices (RFM, BMI, or WC) with all covariates, and compared their gallstone prediction performance using receiver operating characteristic (ROC) curve analysis. Additionally, we performed restricted cubic spline (RCS) analysis to assess the nonlinear relationship between RFM and gallstones. All analyses were conducted utilizing R software version 3.4.3 (R Foundation for Statistical Computing, Vienna, Austria) and EmpowerStats 2.0 software (X&Y Solutions Inc., Boston). Statistical significance was assessed at a two-sided value of *P* < .05.

## 3. Results

### 3.1. Baseline characteristics of participants

Table 1 presents the baseline characteristics of the 5340 participants with a mean age of 50.59 ± 17.14 years, of which 2553 (47.81%) were male and 2787 (52.19%) were female, including 580 (10.86%) with gallstones and 4760 (89.14%) without gallstones. Compared with non-gallstones, gallstones tend to be older, female, non-Hispanic white, of lower height, sugar consumers, smokers, and have diabetes, hypertension, anemia, and asthma (*P* < .05). However, non-gallstones were more likely to have drink intake, a higher education record, and recreational activities compared with gallstones (*P* < .05). Individuals with gallstones were more likely to have higher levels in BMI, WC, and RFM.

**Table 1:** Comparison of baseline characteristics between participants with and without gallstones.

Characteristic	Without gallstones (N = 4760)	With gallstones (N = 580)	*P*-value
Age (yr)	49.76 ± 17.13	57.33 ± 15.67	<.001
PIR	2.68 ± 1.64	2.61 ± 1.56	.491
HDL (mg/dL)	53.63 ± 16.11	52.65 ± 15.17	.236
TC (mg/dL)	186.29 ± 40.77	185.02 ± 43.28	.375
Total energy intake (kcal)	1952.51 ± 951.66	1891.55 ± 877.59	.223
Total sugars (g)	94.59 ± 68.23	100.42 ± 72.43	.011
Total fat (g)	79.70 ± 47.04	78.56 ± 46.53	.592
Water (g)	2639.99 ± 1353.91	2569.95 ± 1241.15	.274
Alcohol (m)	6.90 ± 23.02	4.66 ± 20.70	.004
Dietary fiber (g)	16.27 ± 11.04	15.30 ± 9.37	.338
Caffeine (g)	129.30 ± 181.30	130.79 ± 147.40	.162
RFM	35.88 ± 8.65	41.87 ± 7.80	<.001
Height (cm)	167.35 ± 9.92	164.03 ± 9.03	<.001
WC (cm)	100.73 ± 16.82	109.07 ± 17.51	<.001
BMI (kg/m^2^)	29.80 ± 7.09	33.47 ± 8.45	<.001
Sex (%)			
Male	2391 (50.23%)	162 (27.93%)	<.001
Female	2369 (49.77%)	418 (72.07%)	
Race (%)			
Mexican American	533 (11.20%)	70 (12.07%)	<.001
Other Hispanic	453 (9.52%)	62 (10.69%)	
Non-Hispanic White	1761 (37.00%)	273 (47.07%)	
Non-Hispanic Black	1281 (26.91%)	107 (18.45%)	
Other Race	732 (15.38%)	68 (11.72%)	
Education level (%)			
<9th grade	252 (5.29%)	21 (3.62%)	.008
9–11th grade	486 (10.21%)	65 (11.21%)	
High school graduate	1101 (23.13%)	144 (24.83%)	
Some college or AA degree	1636 (34.37%)	227 (39.14%)	
College graduate or above	1285 (27.00%)	123 (21.21%)	
Hypertension (%)			
Yes	1734 (36.43%)	307 (52.93%)	<.001
No	3026 (63.57%)	273 (47.07%)	
Diabetes (%)			
Yes	651 (13.68%)	147 (25.34%)	<.001
No	4109 (86.32%)	433 (74.66%)	
Asthma (%)			
Yes	750 (15.76%)	118 (20.34%)	.005
No	4010 (84.24%)	462 (79.66%)	
Appropriate recreational activities (%)			
Yes	2019 (42.42%)	209 (36.03%)	.003
No	2741 (57.58%)	371 (63.97%)	
Smoking status (%)			
Yes	1981 (41.62%)	282 (48.62%)	.001
No	2779 (58.38%)	298 (51.38%)	
Anemia (%)			
Yes	482 (10.13%)	83 (14.31%)	.002
No	4278 (89.87%)	497 (85.69%)	

BMI = body mass index, HDL = high density lipoprotein, PIR = ratio of family income to poverty, RFM = relative fat mass, TC = serum total cholesterol, WC = waist circumference.

### 3.2. Association between RFM and gallstones

**Figure 2.**
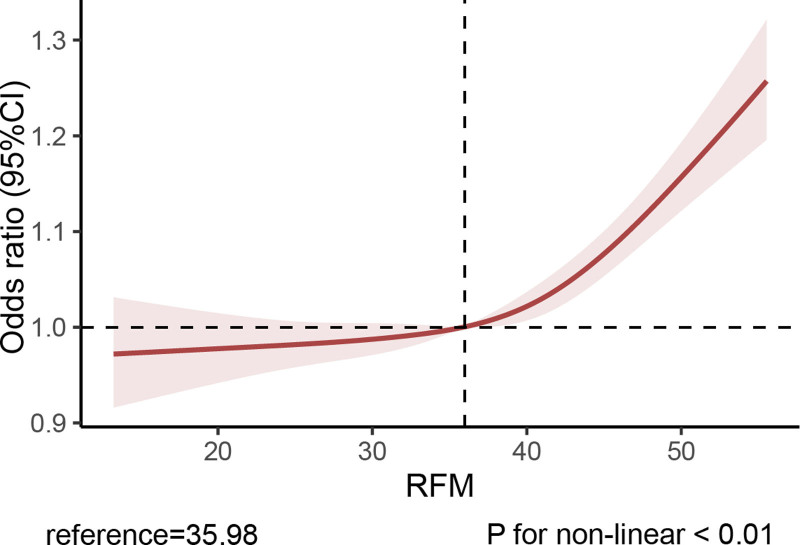
RCS analysis of the association between RFM and gallstones. RCS = restricted cubic spline, RFM = relative fat mass.

The relationship between WC, BMI, RFM, and gallstones is shown in Table 2. The regression analysis showed that a high level of RFM was significantly associated with higher odds of gallstones prevalence. After adjusting for all covariates, the above associations remained significant (odds ratio [OR] = 1.09, 95% confidence interval [CI]: 1.07–1.11), indicating that for each unit of increased RFM, the rate of gallstones increased by 9%. Furthermore, we converted RFM into a 4-categorical variable to conduct sensitivity analysis. Compared with the lowest quartile of RFM, the prevalence of gallstones was increased in the second quartile (OR = 1.62, 95% CI: 1.12–2.34), third quartile (OR = 2.47, 95% CI: 1.60–3.80) and fourth quartile (OR = 4.81, 95% CI: 2.96–7.81), respectively. Between increasing levels of RFM and the odds of gallstones (*P* for trend <.0001). The results of the RCS showed nonlinear association between RFM and gallstones (*P* for non-linear <.01). Further threshold effect analysis indicated that the inflection point for RFM was 35.98. Figure 2 shows the study results.

**Table 2:** Association between WC, BMI, RFM and gallstones.

	Model 1 OR (95% CI)	Model 2 OR (95% CI)	Model 3 OR (95% CI)
WC	1.03 (1.02, 1.03)	1.03 (1.03, 1.04)	1.03 (1.02, 1.03)
BMI	1.06 (1.05, 1.07)	1.07 (1.05, 1.08)	1.05 (1.04, 1.07)
RFM	1.09 (1.08, 1.10)	1.11 (1.09, 1.13)	1.09 (1.07, 1.11)
RFM (quartile)			
Quartile 1	Ref	Ref	Ref
Quartile 2	2.10 (1.48, 2.99)	1.82 (1.27, 2.61)	1.62 (1.12, 2.34)
Quartile 3	3.25 (2.33, 4.53)	3.12 (2.05, 4.75)	2.47 (1.60, 3.80)
Quartile 4	7.12 (5.20, 9.75)	7.06 (4.47, 11.14)	4.81 (2.93, 7.81)
*P* for trend	<.0001	<.0001	<.0001

Model 1: no covariates were adjusted; Model 2: adjusted for sex, age and race; Model 3: adjusted for sex, age, race, education level, PIR, HDL, TC, hypertension, diabetes, asthma, anemia, total energy intake, total fat, total sugars, water, alcohol, dietary fiber, caffeine, appropriate recreational activities, smoking status.

95% CI = 95% confidence interval, BMI = body mass index, HDL = high density lipoprotein, OR = odds ratio, PIR = ratio of family income to poverty, RFM = relative fat mass, TC = serum total cholesterol, WC = waist circumference.

### 3.3. Subgroup analysis

To determine if the relationship between RFM and gallstones remains consistent across the general population and to explore potential subgroup variations, we performed stratified analyses and interaction testing based on age, sex, race, smoking status, hypertension and diabetes. The results of this study showed that the association between RFM and gallstones was only a significant interaction between age (*P* for interaction = .0020), whereas there was no statistically significant association between sex, race, smoking status, hypertension and diabetes (all *P* for interaction > .05). Furthermore, RFM with gallstones remained positively associated in aged 20 to 39 years, aged 40 to 59 years, all sex subgroups, all hypertension, smoking status and diabetes subjects (Table 3).

**Table 3:** Subgroup analyses stratified by age, sex, race, smoking status, hypertension and diabetes.

RFM	Quartile 1 (10.09–30.09)	Quartile 2 (30.09–35.88)	*P*	Quartile 3 (35.88–43.88)	*P*	Quartile 4 (43.89–58.24)	*P*	*P* for interaction
Age								
20–39	Ref	2.12 (0.65, 6.96)	.21	9.55 (2.91, 31.34)	.0002	16.58 (4.60, 59.77)	<.0001	.0020
40–59	Ref	1.47 (0.75, 2.86)	.26	3.53 (1.66, 7.50)	.001	6.33 (2.69, 14.90)	<.0001	
≥60	Ref	1.39 (0.85, 2.26)	.18	0.97 (0.51, 1.86)	.93	1.99 (0.97, 4.11)	.062	
Sex								
Male	Ref	1.67 (1.12, 2.49)	.012	2.01 (1.16, 3.47)	.013	2.35 (1.17,4.14)	.019	.10
Female	Ref	2.27 (0.29, 17.92)	.44	4.78 (0.64, 35.59)	.13	8.87 (1.19, 66.05)	.033	
Race								
Mexican American	Ref	3.82 (0.46, 31.56)	.21	3.69 (0.33, 40.72)	.29	7.26 (0.62, 85.46)	.11	.45
Other Hispanic	Ref	0.68 (0.23, 1.96)	.47	0.94 (0.25, 3.51)	.92	0.90 (0.20, 4.04)	.89	
Non-Hispanic White	Ref	1.64 (0.94, 2.86)	.08	2.56 (1.35, 4.84)	.003	4.37 (2.13, 8.97)	<.0001	
Non-Hispanic Black	Ref	2.46 (1.01, 5.98)	.04	4.71 (1.72, 12.88)	.003	12.35 (3.97, 38.37)	<.0001	
Other Race	Ref	2.16 (0.89, 5.24)	.09	2.76 (0.84, 9.10)	.09	9.21 (2.41, 35.21)	.001	
Smoking status								
Yes	Ref	1.45 (0.90, 2.34)	.12	2.56 (1.44, 4.53)	.013	4.43 (2.27, 8.62)	<.0001	.19
No	Ref	2.17 (1.21, 3.90)	.01	2.73 (1.40, 5.35)	.003	5.79 (2.79, 12.03)	<.0001	
Hypertension								
Yes	Ref	1.25 (0.74, 2.11)	.40	2.00 (1.10, 3.64)	.024	3.87 (1.92, 7.82)	<.001	.34
No	Ref	1.97 (1.17, 3.31)	.01	2.74 (1.44, 5.18)	.002	5.38 (2.70, 10.69)	<.0001	
Diabetes								
Yes	Ref	2.22 (0.91, 5.40)	.08	2.46 (0.93, 6.47)	.07	5.76 (1.73, 19.10)	.004	.94
No	Ref	1.50 (1.00, 2.26)	.05	2.44 (1.47, 4.04)	.006	4.67 (2.70, 8.06)	<.0001	

Adjusted for sex, age, race, education level, high density lipoprotein, serum total cholesterol, ratio of family income to poverty hypertension, diabetes, asthma, anemia, total energy intake, total fat, total sugars, water, alcohol, dietary fiber, caffeine, appropriate recreational activities, smoking status.

RFM = relative fat mass.

### 3.4. Comparison of RFM, BMI, and WC in predicting gallstones

**Figure 3.**
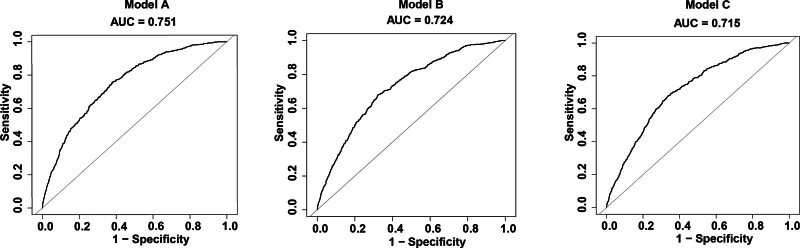
Areas under the ROC curves for the 3 gallstones prediction models in the study data. ROC = receiver operating characteristic.

In Model 3, all indicators associated with obesity are significantly correlated with gallstones (*P* < .05). The odds of gallstones increased by 3% and 5% for each 1 SD increase in WC (OR = 1.03, 95% CI: 1.02–1.03) and BMI (OR = 1.05, 95% CI: 1.04–1.07), the rate of gallstones development increased by 9% for each 1 SD increase in RFM (OR = 1.09, 95% CI: 1.07–1.11) (Table 2). Furthermore, the results of the ROC curves are shown in Figure 3 and Table 4. ROC analysis showed that RFM-integrated (0.751) had a better AUC value than BMI-integrated (0.724) and WC-integrated (0.715) in predicting gallstones. All models adjusted for sex, age, race, education level, high density lipoprotein, serum total cholesterol, ratio of family income to poverty hypertension, diabetes, asthma, anemia, total energy intake, total fat, total sugars, water, alcohol, dietary fiber, caffeine, appropriate recreational activities, smoking status.

## 4. Discussion

This cross-sectional study evaluated the relationship between RFM and gallstones in the American population. Our study found that people with high levels of RFM were prone to gallstones, and the nonlinear positive correlation between RFM and gallstones remained stable in a fully adjusted covariates model. RCS analysis demonstrated a nonlinear association between RFM and gallstones (*P* for non-linear < .01). Threshold effect analysis indicated that the inflection point for RFM was 35.98. In addition, we performed subgroup analyses to confirm the reliability of the results. To evaluate the predictive performance of adiposity measures for gallstones, we conducted ROC curve analyses comparing 3 models: an RFM-based model, a BMI-based model, and a WC-based model. We found that RFM had a better predictive ability for gallstones than BMI and WC.

Numerous epidemiologic studies have shown that obesity is a significant risk factor for the development of gallstones.^[10,32,33]^ Obesity is commonly measured by BMI and WC.^[34,35]^ A recent study by Alyahyawi et al discovered a causal link between BMI and gallstones.^[36]^ In a study conducted in China, elevated BMI and WC were identified as independent risk factors for newly diagnosed gallstones.^[37]^ For every 5% points higher BMI, the risk of gallstone formation rises by 1.63 times, according to a meta-analysis.^[38]^ Furthermore, a Mendelian randomization study confirmed that elevated BMI is a causal risk factor for symptomatic gallstone disease.^[39]^ Moreover, a cross-sectional study found that high WC is the most important factor in gallstone risk [OR = 3.84, 95% CI: 2.11–7.00].^[40]^ In addition, recent studies identified novel obesity indicators associated with gallstones. A study by Park et al showed that weight-adjusted WC index (WWI) not only showed a significant association with gallstones, but also exhibited superior clinical applicability compared to BMI.^[41]^ Likewise, a cross-sectional study found that higher WWI values were positively linked to cholesterol disease.^[33]^ Other commonly used indicators for measuring obesity include waist height ratio (WHtR), waist hips (WHR), body circle index (BRI), etc.^[10,42]^ RFM was a new obesity measure created by researchers in the United States that provided a more precise estimation of total body fat percentage in both sexes compared to BMI,^[22]^ which was consistent with our findings.

According to some previous studies, obesity may increase the risk of gallstones through several pathophysiologic processes. First, obese people consume more high-fat and high-cholesterol foods, leading to supersaturation of cholesterol in bile and a decrease in the synthesis and secretion of bile acids that precipitate as cholesterol gallstones.^[43]^ Second, insulin resistance is one of the key mechanisms underlying obesity or metabolic syndrome, which is also associated with gallstone formation.^[44]^ Research indicated that insulin resistance decreased the expression of bile acid synthesis enzymes, leading to increased cholesterol-saturated bile, which affects gallbladder function and consequently contributes to the development of gallstones.^[45]^ Additionally, elevated GLP-1 secretion following bariatric surgery – similar to the effects of GLP-1 analogues – has been linked to gallbladder hypomotility and increased risk of gallstone formation.^[46]^ Leptin, a hormone released by adipose tissue, is implicated in the formation of gallstones, particularly during obesity. Obesity leads to increased leptin levels, which can raise cholesterol content in bile and elevate the risk of gallstone development.^[47,48]^ Additionally, mouse studies demonstrated that pharmacologic doses of leptin simultaneously promoted weight loss and contributed to cholesterol gallstone formation.^[49]^

Our study had several strengths: First, we used a large representative sample of the U.S. population. Second, RFM is easier to assess than DXA and does not involve radiation, making it easier to use in practice. Third, this was the first study to explore the association between BMI, WC, RFM, and gallstones, and RFM has better potential to predict gallstones than BMI and WC. However, our research had several limitations. First, we were unable to determine a causal association between RFM and gallstones because of the design of the cross-sectional analysis. Second, NHANES data on gallstones were collected through patient questionnaires rather than imaging studies. Since ultrasound examinations are typically performed only for symptomatic cases in clinical practice, our data likely overrepresented symptomatic gallstone cases while potentially missing a substantial number of asymptomatic ones. In addition, we could not incorporate data on all the covariates influencing gallstones, including prior duodenal bypass surgeries, genetic predisposition, medications, and hormonal influences. Finally, the data from the study are specific to the U.S. population and may not be applicable to other countries.

To further validate our findings, future prospective cohort studies with longer follow-up periods are needed to establish the causal relationship between RFM and gallstone development. Additionally, well-designed intervention studies could help determine whether reducing RFM through lifestyle modifications or medical interventions can effectively prevent gallstone formation.

## 5. Conclusions

RFM was positively associated with gallstones in the U.S. adults, particularly in those under 60 years old. Furthermore, RFM has better predictive ability for gallstones compared to BMI and WC.

**Table 4:** **Information of ROC curves in** Figure 3.

Variables	AUC (%)	95% CI (%)	Threshold	Sensitivity	Specificity
RFM	75.06	73.08–77.04	37.15	0.753	0.623
BMI	72.44	70.03–74.54	31.25	0.679	0.677
WC	71.54	69.45–73.64	102.49	0.664	0.663

BMI = body mass index, RFM = relative fat mass, ROC = receiver operating characteristic, WC = waist circumference.

## Acknowledgments

We want to thank all participants in this study.

## Author contributions

**Conceptualization:** Wenshan Zhu, Yucai Jiang.

**Formal analysis:** Yucai Jiang.

**Methodology:** Wenshan Zhu.

**Resources:** Wenshan Zhu.

**Supervision:** Xiaofeng Deng.

**Validation:** Wenshan Zhu, Xiaofeng Deng.

**Visualization:** Yucai Jiang.

**Writing – original draft:** Wenshan Zhu, Yucai Jiang.

**Writing – review & editing:** Wenshan Zhu, Xiaofeng Deng, Yucai Jiang.
